# Convolutional Neural Network-Based Electromagnetic Imaging of Uniaxial Objects in a Half-Space

**DOI:** 10.3390/s25061713

**Published:** 2025-03-10

**Authors:** Chien-Ching Chiu, Jen-Shiun Chiang, Po-Hsiang Chen, Hao Jiang

**Affiliations:** 1 Department of Electrical and Computer Engineering, Tamkang University, Tamsui 251301, Taiwan; chiang@mail.tku.edu.tw (J.-S.C.); 810440031@gms.tku.edu.tw (P.-H.C.); 2 School of Engineering, San Francisco State University, San Francisco, CA 94117-1080, USA; jianghao@sfsu.edu

**Keywords:** artificial intelligence, electromagnetic imaging, uniaxial objects, half-space, convolutional neural network, dominant current scheme, backpropagation scheme

## Abstract

In this paper, we adopt artificial intelligence (AI) technology for the electromagnetic imaging of uniaxial objects buried in a half-space environment. The limited measurement angle inherent to half-space configurations significantly increases the difficulty of data collection. This paper discusses the simultaneous emission of Transverse Magnetic (TM) and Transverse Electric (TE) electromagnetic waves to illuminate a uniaxial object embedded in a half-space. The dominant current scheme (DCS) and the backpropagation scheme (BPS) are subsequently employed to compute the initial permittivity distribution, which is then used as a dataset for training Convolutional Neural Networks (CNNs). The numerical results compare the reconstruction capabilities of both methods under identical conditions, demonstrating that the DCS exhibits superior generalization and noise immunity compared to the BPS. These findings confirm the effectiveness of both schemes in reconstructing the dielectric constant distribution of uniaxial objects buried in a half-space.

## 1. Introduction

Electromagnetic imaging, being an inverse scattering problem, faces challenges such as nonlinearity and non-uniqueness. Electromagnetic sensing technology serves as a powerful tool for identifying the shapes and material of unknown objects by analyzing the scattering of the electromagnetic waves. When combined with the Internet of Things (IoT), it enhances capabilities in sensing, communication systems, and data collection. This synergy drives the development of intelligent and automated systems with applications in diverse domains, such as industrial automation and smart cities [1,2,3,4,5]. Traditional methods like the Born Approximation [6] and its variants [7] provide solutions based on simplified linear models. However, these methods are highly dependent on prior information and battle to surmount inherent nonlinearity and ill-posedness problems [8]. To overcome these challenges, researchers have explored more robust iterative algorithms tailored to full-wave models. Prominent examples include the Distorted Born Iterative Method [9], Source Inversion [10], and the Subspace-based Optimization Method [11]. These advanced techniques deliver high-quality reconstructions but often increase computational complexity and time demands. While iterative algorithms have been employed to address these issues, they are computationally expensive and time-consuming.

Inverse scattering problem (ISP) algorithms are primarily developed to establish nonlinear relationships between the scattered field and the unknown material properties of scatterers. Neural networks have shown considerable promise in building such relationships, although these methods usually rely on pre-existing information about the scatterers. Recently, the exceptional representational power of Deep Neural Networks (DNNs) has spurred their uptake for solving ISPs, with Convolutional Neural Networks (CNNs) playing a particularly prominent role [12,13,14,15,16,17,18,19,20,21,22,23,24,25,26,27]. Deep learning-based methods for ISPs can generally be divided into two main approaches. The first approach seeks to substitute the computationally demanding aspects of traditional nonlinear iterative techniques with trained neural models, thereby simplifying the process [12,13,14]. The second approach frames the ISP as an image-to-image transformation task [15,16,17,18,19,20,21,22,23,24,25,26,27]. A non-iterative physical method is used to generate an initial guess based on the scattered field, and then a CNN is used to refine the generated initial guess into a high-resolution electromagnetic image. This paper emphasizes the second approach, analyzing its potential for delivering superior reconstructions. The second approach showcases the capability of neural networks to generate high-resolution reconstructions, although it remains in its nascent stages. In recent years, significant efforts have been directed toward enhancing imaging quality, with researchers concentrating on optimizing the objective function and integrating regularization techniques to improve both the precision and resilience of the reconstruction process. In 2019, Wei proposed a CNN to tackle the ISP more efficiently. Three training schemes were compared: direct inversion, the BPS, and the DCS. The numerical results showed that the DCS demonstrated superior accuracy and was capable of solving typical ISPs in about 1 s and that the CNN was a promising approach for real-time quantitative imaging [15]. In 2020, Zhang utilized a CNN to reconstruct high-resolution dielectric targets. This approach combined a qualitative Direct Sampling Method (DSM) with a quantitative backpropagation (BP) technique to capture spatial information for preliminary parameter estimation. The proposed method improved the reconstruction quality of the U-Net CNN without increasing computational complexity [16]. In 2021, Zhou introduced the Modified Contrast Scheme (MCS) to address the nonlinear ISP. This scheme leveraged local wave amplifier coefficients to create a modified contrast ratio, which helped to reduce the overall nonlinearity while maintaining accuracy. The numerical results indicated that after offline training, the MCS was effective in both two-dimensional and three-dimensional environments, particularly showing significant improvements in reconstructing high-contrast scatterers [17]. In 2022, Song proposed a unified learning-based approach to solve the ISP involving mixed boundary conditions. He first used a T-matrix method to simulate the scattering behavior of mixed dielectrics and Perfect Electric Conductors (PECs). Next, he reconstructed a rough image of the unknown scatterer using the backpropagation method, which was then enhanced by an attention-assisted pix2pix Generative Adversarial Network (GAN). Numerical tests validated the effectiveness of this method in reconstructing scatterers without any prior knowledge of their boundary conditions [18]. In 2023, Wang introduced a novel Early Fusion Framework (EFF) designed to address the ISP. The EFF architecture integrated a Scattered Field Encoder (SFE) and a Backpropagation Encoder (BPE). The SFE primarily resolved the nonlinear relationships arising from multiple scattering, while the BPE offered prior information based on the pre-reconstructed backpropagated distribution. The numerical results demonstrated that the EFF exhibited excellent accuracy, stability, and generalization capability [19]. In 2024, Wu introduced a method called Contrastive Learning-Based Subspace Optimization and Semantic Segmentation Assisted Reconstruction (CLSO-SSR). This network improved the initial estimation of contrasting sources, which helped reduce the likelihood of reaching local optima and decreased the time needed for inversion. It also incorporated a semantic segmentation network with an attention mechanism to address the nonlinearities arising from multiple scattering effects. Numerical experiments demonstrated the effectiveness of CLSO-SSR in estimating dielectric constants [20].

Recent studies have made significant advancements in electromagnetic imaging technology within half-space scenarios. These research studies are focused on enhanced methodologies and applications that leverage the unique challenges posed by the half-space environment. In 2019, Chiu used Transverse Magnetic (TM) polarized waves to illuminate a periodical rough surface and applied the Self-Adaptive Dynamical Differential Evolution (SADDE) algorithm to tackle the inverse scattering problem. The results indicated that SADDE effectively converged to the global extrema. Additionally, the period length exhibited a significantly faster convergence speed compared to the shape function and dielectric constant [21]. In 2023, Topbaş developed a hybrid method for locating cylinders buried in a half-space with a known orientation. This approach combined the classical moment method applied to the vertical coordinate with a spectral expansion for the horizontal coordinate. It utilized Green’s function and Tikhonov regularization to address the problem’s ill-posedness. An example was provided to demonstrate the method’s applicability and accuracy [22]. In the same year, Chiu proposed a Deep Convolutional Neural Network to reconstruct the shape of the conductor. The measured scattered field was used as the direct input of the neural network to reconstruct the shape of the conductor in a half-space [23]. In 2024, Chiu introduced a novel artificial intelligence technique that incorporated an attention mechanism for half-space electromagnetic imaging. The BPS generated an initial guess of the image for the scatterer located in the lower half-space based on the measured scattered field. These guessed images were then input into a Self-Attention Generative Adversarial Network (SAGAN) for reconstruction. The numerical results demonstrated that SAGAN had effectively reconstructed the electromagnetic images with high accuracy [24].

**Figure 1:**
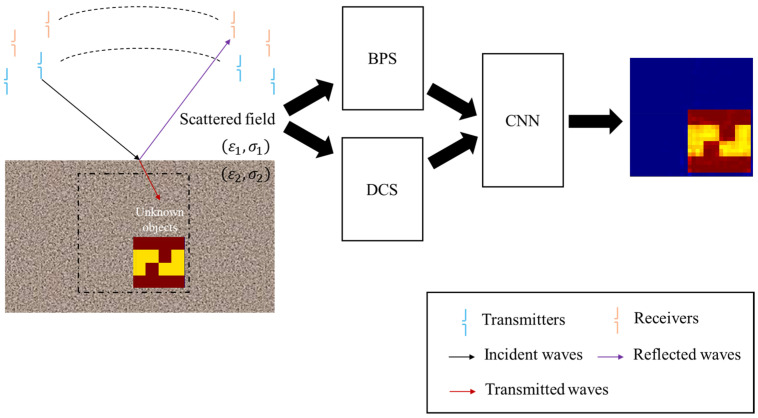
Electromagnetic sensing architecture.

The architecture of our system is depicted in Figure 1. In this setup, we strategically place transmitters within the simulation environment to illuminate the target object while receivers capture the scattered field generated. We then utilize the gathered scattered field data to produce an initial image using the BPS and the DCS. These initial images are subsequently processed by a CNN for image reconstruction, transforming the estimated images into high-resolution electromagnetic images. This research makes several significant contributions:

1. To date, there are no existing publications addressing the electromagnetic imaging of buried uniaxial objects in a half-space using artificial intelligence technology. We introduce a novel approach that integrates the DCS with a CNN to tackle the complex nonlinear inverse scattering problem.
2. Given that measurements are restricted to the upper space, the range of measuring angles is inherently limited. Our numerical simulations demonstrate that the proposed method effectively images highly nonlinear scatterers with both speed and accuracy while exhibiting robust noise immunity.
3. The dielectric constant vector sum derived from the electric field of Transverse Electric (TE) polarized waves manifests as a tensor across the cross-section, presenting greater complexity compared to the dielectric constant vector sum associated with Transverse Magnetic (TM) polarized waves. The interaction between the dielectric constant tensor and the electric field results in strong directional dependence in the TE polarized waves, leading to difficult reconstruction.
4. A uniaxial scatterer possesses dielectric constant components that vary with direction. The nonlinear characteristics of the TE polarized waves present considerably more challenges than their TM counterparts, complicating the reconstruction process using the scattered fields.
5. We successfully amalgamate the DCS with CNN methodologies to reconstruct electromagnetic images of buried objects in a half-space environment. Our numerical analyses reveal the reconstruction efficacy of this combined approach. To validate the robustness of our method, we employ a pretrained model to reconstruct scenarios involving high dielectric constant distributions. The results confirm that our technique maintains high reliability, even in half-space settings.

In Section 2, we provide a theoretical derivation of the TM and TE modes. Section 3 focuses on utilizing the U-Net architecture to reconstruct images from uniaxial objects. Section 4 presents numerical results and simulation analyses, comparing the outcomes from the U-Net reconstruction with the initial estimates obtained from the DCS and BPS techniques. This comparison is made under various noise conditions while maintaining consistent deep learning training parameters and network architecture. Finally, Section 5 summarizes the findings and conclusions drawn from our research.

## 2. Theory

### 2.1. Direct Problems

**Figure 2:**
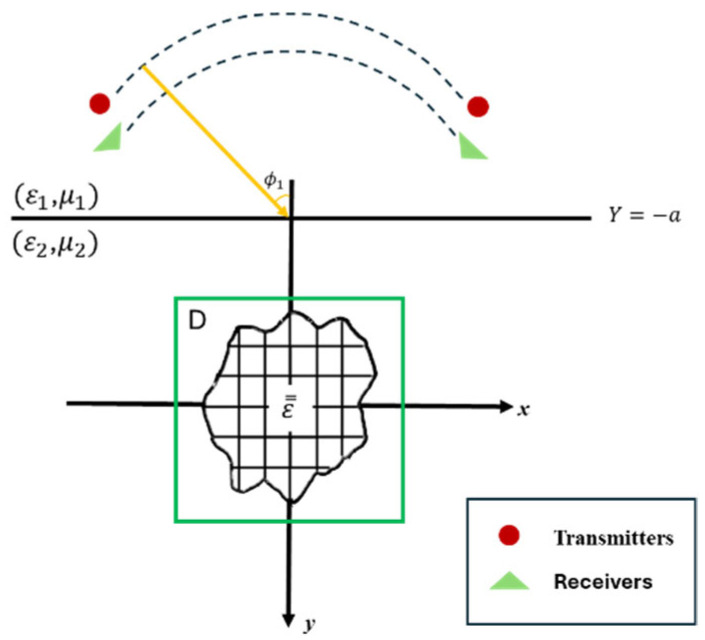
Schematic diagram of a two-dimensional object located in a lossy half-space.

Assume a uniaxial object ${\overset{̿}{\varepsilon }}_{r}$ is buried in a lossy half-space, as shown in Figure 2. $\varepsilon_{1}$ and $\varepsilon_{2}$ represent the dielectric constants of Region 1 and Region 2, respectively. Similarly, $\sigma_{1}$ and $\sigma_{2}$ signify the conductivity of Region 1 and Region 2, respectively. Let $\mu_{0}$ be the permeability of free space in each region. In other words, this article focuses exclusively on scatterers as nonmagnetic materials. We begin by introducing a dielectric object in which the scatterer is infinitely extended along the z-axis. Specifically, we consider an incident wave with TM polarization. We define the relationship between the incident wave and its time variation as $e^{j\omega t}$, and we denote its angle of incidence as $\emptyset_{1}$.

In the context of the Cartesian coordinate system, which consists of the axes x, y, and z, we can define a diagonal matrix for permittivities, as illustrated in Equation (1). $\varepsilon_{x}(x,y)$, $\varepsilon_{y}(x,y)$, and $\varepsilon_{z}(x,y)$ are typically represented as complex values.

(1) $${\overset{̿}{\varepsilon }=\left[\begin{array}{ccc} \varepsilon_{x}\left(x,y\right) & 0 & 0\\ 0 & \varepsilon_{y}\left(x,y\right) & 0\\ 0 & 0 & \varepsilon_{z}\left(x,y\right) \end{array}\right]}_{xyz}$$

To make our analysis clearer and more accurate, we investigate the TM wave first, which is aligned parallel to the z-axis. We designate the electric field in a medium without any scatterers as $E_{z}^{i}$. We can describe this electric field with Equation (2), which shows how it behaves and distributes under the given conditions. This method helps us focus on the key parts of wave propagation and interaction, without being distracted by the effects of scatterers.

(2) $$E_{z}^{i}\left(x,y\right)=\left\{\begin{array}{cc} E_{1}\left(x,y\right)=e^{-jk_{1}\left[x\sin\emptyset_{1}+\left(y+a\right)\cos\emptyset_{1}\right]}+{\frac{1-n^{TM}}{1+n^{TM}}e}^{-jk_{1}\left[x\sin\emptyset_{1}-\left(y+a\right)\cos\emptyset_{1}\right]} & ,\;y\leq -a\\ E_{2}\left(x,y\right)=\frac{2}{1+n^{TM}}e^{-jk_{2}\left[x\sin\emptyset_{2}+\left(y+a\right)\cos\emptyset_{2}\right]} & ,\;y\geq -a \end{array}\right.$$

where $n^{TM}=\frac{{cos\emptyset }_{2}}{{cos\emptyset }_{1}}\sqrt{\frac{{\varepsilon_{0}\varepsilon }_{2}-j\sigma_{2}/\omega }{\varepsilon_{0}\varepsilon_{1}-j\sigma_{1}/\omega }}$ and $k_{1}sin\emptyset_{1}=k_{2}sin\emptyset_{2}$.

(3) $$k_{i}^{2}=\omega^{2}\varepsilon_{0}\varepsilon_{i}\mu_{0}-j\omega \mu_{0}\sigma_{i}\;,\;i=1,2\;Im(k_{i})\leq 0$$

In cases where Regions 1 and 2 consist of lossless materials that do not absorb energy as electromagnetic waves pass through them, $\emptyset_{1}$ and $\emptyset_{2}$ represent the angles of incidence and refraction, respectively. However, when Regions 1 and 2 contain lossy materials that dissipate energy as heat or through other mechanisms, the behavior of the angles $\emptyset_{1}$ and $\emptyset_{2}$ becomes more complex. In these situations, the interaction of waves with the lossy materials may introduce additional factors, such as attenuation and phase shifts. This complexity necessitates the implementation of more advanced models to accurately describe the angles and the associated wave propagation. Therefore, analyzing angles in lossy media requires a deeper understanding of how energy loss influences the overall behavior of the incident and refracted waves. Based on the principles of induced current and Maxwell’s equations, we can derive the following relationships:

(4) $$\nabla \times \overset{⃑}{E}=-j\omega \mu_{0}\overset{⃑}{H}$$

(5) $$\nabla \times \overset{⃑}{H}=j\omega \varepsilon_{0}\varepsilon_{2}\overset{⃑}{E}+{\overset{⃑}{j}}_{eq}$$

where ${\overset{⃑}{j}}_{eq}=j\omega \varepsilon_{0}\left[\varepsilon_{z}\left(x,y\right)-\varepsilon_{2}\right]E\hat{z}$ is the equivalent current density of the dielectric object.

In this context, ${\overset{⃑}{j}}_{eq}=j\omega \varepsilon_{0}\left[\varepsilon_{z}\left(x,y\right)-\varepsilon_{2}\right]E^{t}\hat{z}$ represents the equivalent current density of the dielectric object. The total electric field within the object, where ${\overset{⃑}{E}}_{z}^{t}\left(x,y\right)=E_{z}^{t}\left(x,y\right)\hat{z}=\left[E_{z}^{i}\;\left(x,y\right)+E_{z}^{s}\left(x,y\right)\right]\hat{z}$ can be expressed using the two-dimensional Green’s function as:

(6) $$E_{z}^{i}\;\left(\bar{r}\right)=\int_{s}G\left(r,r^{\prime }\right)k_{2}^{2}\left[\varepsilon_{r}\left(r^{\prime }\right)-\varepsilon_{2}\right]E_{z}^{t}\left(r^{\prime }\right)ds^{\prime },\;y>-a$$

The scattered field can be written as:

(7) $$E_{z}^{s}\left(\bar{r}\right)=-\int_{s}G\left(r,r^{\prime }\right)k_{2}^{2}\left[\varepsilon_{r}\left(r^{\prime }\right)-\varepsilon_{2}\right]E_{z}^{t}\left(r^{\prime }\right)ds^{\prime }$$

To solve the half-space issue, we need to solve for the Green’s function first, denoted as G(x, y; x′, y′). This requires using a line current source located at a specific point and determining the scattered field at another point. By employing the Fourier transform technique, the half-space Green’s function can be expressed as:

(8) $$G\left(x,y{;x}^{\prime },y^{\prime }\right)=\left\{\begin{array}{ll} G_{1}\left(x,y{;x}^{\prime },y^{\prime }\right) & ,\;y\leq -a\\ G_{2}\left(x,y{;x}^{\prime },y^{\prime }\right)=G_{f}\left(x,y{;x}^{\prime },y^{\prime }\right)+G_{s}\left(x,y{;x}^{\prime },y^{\prime }\right) & ,y>-a \end{array}\right.$$

(9) $$G_{1}\left(x,y{;x}^{\prime },y^{\prime }\right)=\frac{1}{2\pi }\int_{-\infty }^{\infty }\frac{j}{\gamma_{1}+\gamma_{2}}e^{j\gamma_{1}\left(y+a\right)}e^{-j\gamma_{2}\left(y\prime +a\right)}e^{-j\alpha \left(x-x^{\prime }\right)}d\alpha$$

(10a) $$G_{f}\left(x,y{;x}^{\prime },y^{\prime }\right)=\frac{j}{4}H_{0}^{\left(2\right)}\left[k_{2}\sqrt{(x-x\prime {)}^{2}+(y-y\prime {)}^{2}}\right]$$

(10b) $$G_{s}\left(x,y{;x}^{\prime },y^{\prime }\right)=\frac{1}{2\pi }\int_{-\infty }^{\infty }\frac{j}{2\gamma_{2}}\left(\frac{\gamma_{2}-\gamma_{1}}{\gamma_{2}+\gamma_{1}}\right)e^{-j\gamma_{2}\left(y+2a+y^{\prime }\right)}e^{-j\alpha \left(x-x^{\prime }\right)}d\alpha$$

(11) γi2=ki2−α2, i=1,2, Im(γi)≤0

In this context, $k_{i}$ represents the wave number for the *i*-th region, while G(x, y; x′, y′) denotes the half-space Green’s function obtained through the Fourier transform. Here, $H_{0}^{2}$ refers to the second-order zero Hankel function. Accurately calculating the Green’s function in Equation (8) is essential for the numerical solutions of Equations (9) and (10). However, when the points (x, y) and (x′, y′) are near the interface between the two regions at y=−a, the integration process will converge slowly. This leads to a significant computational burden when evaluating the half-space Green’s function in such scenarios.

In this paper, the electric field for TE waves propagates along the x-axis and y-axis in a half-space. In a homogeneous medium without scatterers, we can use Equation (12) to represent the electric field associated with these TE waves. The component of the electric field along the x-axis is denoted as $E_{x}^{i}$, while the component along the y-axis is represented as $E_{y}^{i}$. By expressing the overall electric field in terms of these components, we can analyze the behavior of the fields in relation to one another.

(12) $$E_{xy}^{i}(x,y)=\left\{\begin{array}{cc} E_{3}\left(x,y\right)=\left[-\hat{x}\cos\phi_{1}+\hat{y}\sin\phi_{1}\right]e^{-jk_{1}\left[x\sin\emptyset_{1}+\left(y+a\right)\cos\emptyset_{1}\right]}+\left[\hat{x}\cos\phi_{1}+\hat{y}\sin\phi_{1}\right]{\frac{1-n^{TE}}{1+n^{TE}}e}^{-jk_{1}\left[x\sin\emptyset_{1}-\left(y+a\right)\cos\emptyset_{1}\right]} & ,y\leq -a\\ E_{4}\left(x,y\right)=\left(-\hat{x}\cos\phi_{2}+\hat{y}\sin\phi_{2}\right)\frac{\eta_{2}}{\eta_{1}}\frac{2}{1+n^{TE}}e^{-jk_{2}\left[x\sin\emptyset_{2}+\left(y+a\right)\cos\emptyset_{2}\right]} & ,y>-a \end{array}\right.$$

(13) $$n^{TE}=\frac{cos\emptyset_{2}}{cos\emptyset_{1}}\sqrt{\frac{{\varepsilon_{0}\varepsilon }_{1}-j\sigma_{1}/\omega }{\varepsilon_{0}\varepsilon_{2}-j\sigma_{2}/\omega }},\eta_{1}=\sqrt{\frac{\mu_{0}}{{\varepsilon_{0}\varepsilon }_{1}-j\sigma_{1}/\omega }},\;\eta_{2}=\sqrt{\frac{\mu_{0}}{\varepsilon_{0}\varepsilon_{2}-j\sigma_{2}/\omega }}$$

where $E_{3}$ represents the direct wave of the electromagnetic wave irradiating Region 1, as well as the reflected wave from the uniform medium. $E_{4}$ represents the refracted wave of the electromagnetic wave incident on Region 2 through Region 1. The integral equation of the total electric field is more complex to analyze due to the coupling effect between $E_{x}$ and *E_y_*, as described by Maxwell’s equations.

(14) $$\nabla \times \overset{⃑}{E}=-j\omega \mu_{0}\overset{⃑}{H}$$

(15) $$\nabla \times \overset{⃑}{H}=j\omega {\varepsilon_{0}\varepsilon }_{2}\overset{⃑}{E}+{\overset{⃑}{J}}_{eq}$$

where ${\overset{⃑}{J}}_{eq}=j\omega \varepsilon_{0}[\varepsilon_{x}(x,y)-\epsilon_{2}]E_{x}\hat{x}+j\omega \varepsilon_{0}[\varepsilon_{y}(x,y)-\epsilon_{2}]E_{y}\hat{y}$ denotes the equivalent current density in the medium. The total field and scattered field are given by the following Equations (16)–(19):

$$E_{x}\left(x,y\right)=-\left(\frac{\partial^{2}}{\partial x^{2}}+k_{2}^{2}\right)\left[\int_{\begin{array}{c} s \end{array}}G\left(x,y{;x}^{\prime },y^{\prime }\right)\left(\frac{\varepsilon_{x}\left(x^{\prime },y^{\prime }\right)}{\epsilon_{2}}-1\right)E_{x}\left(x^{\prime },y^{\prime }\right)ds^{\prime }\right]$$

(16) $$-\frac{\partial^{2}}{\partial x\partial y}\left[\int_{\begin{array}{c} s \end{array}}G(x,y{;x}^{\prime },y^{\prime })(\frac{\varepsilon_{y}\left(x^{\prime },y^{\prime }\right)}{\epsilon_{2}}-1)E_{y}(x^{\prime },y^{\prime })ds\prime \right]+E_{x}^{i}(x,y)$$

$$E_{y}\left(x,y\right)=-\frac{\partial^{2}}{\partial x\partial y}\left[\int_{\begin{array}{c} s \end{array}}G\left(x,y{;x}^{\prime },y^{\prime }\right)\left(\frac{\varepsilon_{x}\left(x^{\prime },y^{\prime }\right)}{\epsilon_{2}}-1\right)E_{x}\left(x^{\prime },y^{\prime }\right)ds^{\prime }\right]$$

(17) $$-\left(\frac{\partial^{2}}{\partial y^{2}}+k_{2}^{2}\right)\left[\int_{\begin{array}{c} s \end{array}}G(x,y{;x}^{\prime },y^{\prime })(\frac{\varepsilon_{y}\left(x^{\prime },y^{\prime }\right)}{\epsilon_{2}}-1)E_{y}(x^{\prime },y^{\prime })ds\prime \right]+E_{y}^{i}(x,y)$$

$$E_{x}^{s}\left(x,y\right)=-\left(\frac{\partial^{2}}{\partial x^{2}}+k_{2}^{2}\right)\left[\int_{\begin{array}{c} s \end{array}}G\left(x,y{;x}^{\prime },y^{\prime }\right)\left(\frac{\varepsilon_{x}\left(x^{\prime },y^{\prime }\right)}{\epsilon_{2}}-1\right)E_{x}\left(x^{\prime },y^{\prime }\right)ds^{\prime }\right]$$

(18) $$-\frac{\partial^{2}}{\partial x\partial y}\left[\int_{\begin{array}{c} s \end{array}}G(x,y{;x}^{\prime },y^{\prime })(\frac{\varepsilon_{y}\left(x^{\prime },y^{\prime }\right)}{\epsilon_{2}}-1)E_{y}(x^{\prime },y^{\prime })ds\prime \right]$$

$$E_{y}^{s}\left(x,y\right)=-\frac{\partial^{2}}{\partial x\partial y}\left[\int_{\begin{array}{c} s \end{array}}G\left(x,y{;x}^{\prime },y^{\prime }\right)\left(\frac{\varepsilon_{x}\left(x^{\prime },y^{\prime }\right)}{\epsilon_{2}}-1\right)E_{x}\left(x^{\prime },y^{\prime }\right)ds^{\prime }\right]$$

(19) $$-\left(\frac{\partial^{2}}{\partial y^{2}}+k_{2}^{2}\right)\left[\int_{\begin{array}{c} s \end{array}}G(x,y{;x}^{\prime },y^{\prime })(\frac{\varepsilon_{y}\left(x^{\prime },y^{\prime }\right)}{\epsilon_{2}}-1)E_{y}(x^{\prime },y^{\prime })ds\prime \right]$$

The Green’s functions for the TE wave in a half-space are expressed by the following equations:

(20) $${\left(G_{3}\right)}_{mn}=\left\{\begin{array}{cc} \frac{\partial^{2}G_{s}\left(x,y;x_{n},y_{n}\right)}{\partial x^{2}}\left|\begin{array}{c} x=x_{m}\\ y=y_{m} \end{array}\right.\cdot \Delta S_{n}+G_{s}\left(x_{m},y_{m};x_{n},y_{n}\right)\cdot k_{2}^{2}\cdot \Delta S_{n}+\frac{j\pi a_{n}J_{1}\left(k_{2}a_{n}\right)}{2\rho_{mn}^{3}}\left[k_{2}\rho_{mn}{\left(y_{m}-y_{n}\right)}^{2}H_{0}^{\left(2\right)}\left(k_{2}\rho_{mn}\right)+\left({\left(x_{m}-x_{n}\right)}^{2}-{\left(y_{m}-y_{n}\right)}^{2}\right)H_{1}^{\left(2\right)}\left(k_{2}\rho_{mn}\right)\right] & ,\;m\neq n\\ \frac{\partial^{2}G_{s}\left(x,y;x_{n},y_{n}\right)}{\partial x^{2}}\left|\begin{array}{c} x=x_{m}\\ y=y_{m} \end{array}\right.\cdot \Delta S_{n}+G_{s}\left(x_{m},y_{m};x_{n},y_{n}\right)\cdot k_{2}^{2}\cdot \Delta S_{n}+\frac{j}{4}\left[\pi k_{2}a_{n}H_{1}^{\left(2\right)}\left(k_{2}a_{n}\right)-4j\right] & ,\;m=n \end{array}\right.$$

(21) $${\left(G_{4}\right)}_{mn}=\left\{\begin{array}{cc} \frac{\partial^{2}G_{s}\left(x,y;x_{n},y_{n}\right)}{\partial x\partial y}\left|\begin{array}{c} x=x_{m}\\ y=y_{m} \end{array}\right.\cdot \Delta S_{n}+\frac{j\pi a_{n}J_{1}\left(k_{2}a_{n}\right)}{2\rho_{mn}^{3}}\left(x_{m}-x_{n}\right)\left(y_{m}-y_{n}\right)\left[2H_{1}^{\left(2\right)}\left(k_{2}\rho_{mn}\right)-k_{2}\rho_{mn}H_{0}^{\left(2\right)}\left(k_{2}\rho_{mn}\right)\right] & ,\;m\neq n\\ 0 & ,\;m=n \end{array}\right.$$

(22) $${\left(G_{5}\right)}_{mn}=\left\{\begin{array}{cc} \frac{\partial^{2}G_{s}\left(x,y;x_{n},y_{n}\right)}{\partial y^{2}}\left|\begin{array}{c} x=x_{m}\\ y=y_{m} \end{array}\right.\cdot \Delta S_{n}+G_{s}\left(x_{m},y_{m};x_{n},y_{n}\right)\cdot k_{2}^{2}\cdot \Delta S_{n}+\frac{j\pi a_{n}J_{1}\left(k_{2}a_{n}\right)}{2\rho_{mn}^{3}}\left[k_{2}\rho_{mn}{\left(x_{m}-x_{n}\right)}^{2}H_{0}^{\left(2\right)}\left(k_{2}\rho_{mn}\right)+\left({\left(y_{m}-y_{n}\right)}^{2}-{\left(x_{m}-x_{n}\right)}^{2}\right)H_{1}^{\left(2\right)}\left(k_{2}\rho_{mn}\right)\right] & ,\;m\neq n\\ \frac{\partial^{2}G_{s}\left(x,y;x_{n},y_{n}\right)}{\partial y^{2}}\left|\begin{array}{c} x=x_{m}\\ y=y_{m} \end{array}\right.\cdot \Delta S_{n}+G_{s}\left(x_{m},y_{m};x_{n},y_{n}\right)\cdot k_{2}^{2}\cdot \Delta S_{n}+\frac{j}{4}\left[\pi k_{2}a_{n}H_{1}^{\left(2\right)}\left(k_{2}a_{n}\right)-4j\right] & ,\;m=n \end{array}\right.$$

(23) $${\left(G_{6}\right)}_{mn}=\frac{\partial^{2}G_{1}\left(x,y;x_{n},y_{n}\right)}{\partial x^{2}}\left|\begin{array}{c} x=x_{m}\\ y=y_{m} \end{array}\right.\cdot \Delta S_{n}+G_{1}\left(x_{m},y_{m};x_{n},y_{n}\right)\cdot k_{2}^{2}\cdot \Delta S_{n}$$

(24) $${\left(G_{7}\right)}_{mn}=\frac{\partial^{2}G_{1}\left(x,y;x_{n},y_{n}\right)}{\partial x\partial y}\left|\begin{array}{c} x=x_{m}\\ y=y_{m} \end{array}\right.\cdot \Delta S_{n}$$

(25) $${\left(G_{8}\right)}_{mn}=\frac{\partial^{2}G_{1}\left(x,y;x_{n},y_{n}\right)}{\partial y^{2}}\left|\begin{array}{c} x=x_{m}\\ y=y_{m} \end{array}\right.\cdot \Delta S_{n}+G_{1}\left(x_{m},y_{m};x_{n},y_{n}\right)\cdot k_{2}^{2}\cdot \Delta S_{n}$$

where $\rho_{mn}=\sqrt{{\left(x_{m}-x_{n}\right)}^{2}+{\left(y_{m}-y_{n}\right)}^{2}}$ and $H_{0}^{\left(2\right)}$ is the zero-order Hankel function of the second kind, ${H_{1}}^{\left(2\right)}$ is the first-order Hankel function of the second kind, $J_{1}$ is the first-order Bessel function, ($x_{m},\;y_{m}$) is the m-th observation point, and ($x_{n},\;y_{n}$) is the n-th source point. In the next subsection, we introduce an imaging method that solves the inverse scattering problem. The measured scattered field is used to generate initial guesses for the BPS and the DCS. This process of generating initial guesses can greatly reduce the difficulty of CNN training.

### 2.2. Inverse Problem

Within the framework of the inverse scattering problem, we focus on measuring the scattered field in the external region surrounding the scatterer. Notably, in the TM case, the sole unknown dielectric parameter is $\varepsilon_{z}$, while in the TE case, the unknowns involve the dielectric constants $\varepsilon_{x}$ and $\varepsilon_{y}$. Next, we proceed by applying the method of moments to reformulate the resulting integral equations into a matrix representation. Subsequently, we employ the BPS and the DCS to generate the initial dielectric constant distribution. As a final step, we leverage a U-Net architecture for deep learning to accurately reconstruct the dielectric constant tensor.

#### 2.2.1. BPS

In this section, we leverage the measured scattered field data to derive an initial permittivity distribution via the BPS, aiming to streamline the training process of the U-Net model. Previous studies have shown that the BPS is particularly good at reconstructing weak scatterers. We further postulate that the backpropagation field is directly proportional to the induced currents $I_{z}^{b},\;I_{x}^{b}$, and $I_{y}^{b}$.

(26) $$(I_{z}^{b})=\Upsilon_{m}\cdot {\left[G_{2}\right]}^{H}\left(E_{z}^{s}\right)$$

(27) $$\left(\begin{array}{c} I_{x}^{b}\\ I_{y}^{b} \end{array}\right)=\Upsilon_{e}\cdot {\left[\begin{array}{cc} \left[G_{6}\right] & \left[G_{7}\right]\\ \left[G_{7}\right] & \left[G_{8}\right] \end{array}\right]}^{H}\left(\begin{array}{c} E_{x}^{s}\\ E_{y}^{s} \end{array}\right)$$

where *H* represents the conjugate transpose. The loss function is defined as follows:

(28) $$L_{m}^{b}(\Upsilon )={\left\|\left(E_{z}^{s}\right)-\left[G_{2}\right]\cdot \Upsilon_{m}\cdot {\left[G_{2}\right]}^{H}\left(E_{z}^{s}\right)\right\|}^{2}$$

(29) $$L_{e}^{b}(\Upsilon )={\left\|\left(\begin{array}{c} E_{x}^{s}\\ E_{y}^{s} \end{array}\right)-\left[\begin{array}{cc} \left[G_{6}\right] & \left[G_{7}\right]\\ \left[G_{7}\right] & \left[G_{8}\right] \end{array}\right]\cdot \Upsilon_{e}\cdot {\left[\begin{array}{cc} \left[G_{6}\right] & \left[G_{7}\right]\\ \left[G_{7}\right] & \left[G_{8}\right] \end{array}\right]}^{H}\left(\begin{array}{c} E_{x}^{s}\\ E_{y}^{s} \end{array}\right)\right\|}^{2}$$

To determine the minimum of the loss function, we set its derivative to zero. The resulting analytical expressions for $\Upsilon_{m}$ and $\Upsilon_{e}$ can be derived as follows:

(30) $$\Upsilon_{m}=\frac{{\left(E_{z}^{s}\right)}^{T}\cdot {\left(\left[G_{2}\right]\left({\left[G_{2}\right]}^{H}\cdot \left(E_{z}^{s}\right)\right)\right)}^{*}}{{\left\|\left[G_{2}\right]\left({\left[G_{2}\right]}^{H}\cdot \left(E_{z}^{s}\right)\right)\right\|}^{2}}$$

(31) $$\Upsilon_{e}=\frac{{\left(\begin{array}{c} E_{x}^{s}\\ E_{y}^{s} \end{array}\right)}^{T}\cdot {\left(\left[\begin{array}{cc} \left[G_{6}\right] & \left[G_{7}\right]\\ \left[G_{7}\right] & \left[G_{8}\right] \end{array}\right]\left({\left[\begin{array}{cc} \left[G_{6}\right] & \left[G_{7}\right]\\ \left[G_{7}\right] & \left[G_{8}\right] \end{array}\right]}^{H}\cdot \left(\begin{array}{c} E_{x}^{s}\\ E_{y}^{s} \end{array}\right)\right)\right)}^{*}}{{\left\|\left[\begin{array}{cc} \left[G_{6}\right] & \left[G_{7}\right]\\ \left[G_{7}\right] & \left[G_{8}\right] \end{array}\right]\left({\left[\begin{array}{cc} \left[G_{6}\right] & \left[G_{7}\right]\\ \left[G_{7}\right] & \left[G_{8}\right] \end{array}\right]}^{H}\cdot \left(\begin{array}{c} E_{x}^{s}\\ E_{y}^{s} \end{array}\right)\right)\right\|}^{2}}$$

where *T* and * represent the transpose and complex conjugate, respectively.

The induced current can be derived from Υ, and the total field of BPS can also be determined accordingly. The total electric field $E_{z}^{b}$ is defined as follows:

(32) $$\left(E_{z}^{b}\right)=\left(E_{z}^{i}\right)+\left[G_{1}\right]\left(I_{z}^{b}\right)$$

(33) $$\left(\begin{array}{c} E_{x}^{b}\\ E_{y}^{b} \end{array}\right)=\left(\begin{array}{c} E_{x}^{i}\\ E_{y}^{i} \end{array}\right)+\left[\begin{array}{cc} \left[G_{3}\right] & \left[G_{4}\right]\\ \left[G_{4}\right] & \left[G_{5}\right] \end{array}\right]\left(\begin{array}{c} I_{x}^{b}\\ I_{y}^{b} \end{array}\right)$$

The correlation between the induced current and contrast can be expressed as:

(34) $$\left(I_{z,p}^{b}\right)=diag\left(\left[\tau_{z}^{b}\right]\right)\left(E_{z}^{b}\right)$$

(35) $$\left(\begin{array}{c} I_{x,p}^{b}\\ I_{y,p}^{b} \end{array}\right)=diag\left(\left[\begin{array}{cc} \left[\tau_{x}^{b}\right] & 0\\ 0 & \left[\tau_{y}^{b}\right] \end{array}\right]\right)\left(\begin{array}{c} E_{x}^{b}\\ E_{y}^{b} \end{array}\right)$$

where *p* represents the number of incidences and $\tau^{b}$ represents the distribution of dielectric coefficients during BP. By aggregating all instances of Equations (34) and (35) and applying the least squares method, we can derive an analytical expression for the *n*-th element of the contrast $\tau^{b}$ as follows:

(36) $$\left[\tau_{z}^{b}\right]=\frac{\sum_{p=1}^{Ni}I_{z,\;p}^{b}\left(n\right)\cdot {\left[E_{z}^{b}\left(n\right)\right]}^{*}}{\sum_{p=1}^{Ni}{\left\|E_{z}^{b}\left(n\right)\right\|}^{2}}$$

(37) $$\left[\begin{array}{cc} \left[\tau_{x}^{b}\right] & 0\\ 0 & \left[\tau_{y}^{b}\right] \end{array}\right]=\frac{\sum_{p=1}^{Ni}\begin{array}{c} I_{x,p}^{b}\left(n\right)\\ I_{y,p}^{b}\left(n\right) \end{array}\cdot {\left[\left(\begin{array}{c} E_{x}^{b}\\ E_{y}^{b} \end{array}\right)\left(n\right)\right]}^{*}}{\sum_{p=1}^{Ni}{\left\|\begin{array}{c} E_{x}^{b}\left(n\right)\\ E_{y}^{b}\left(n\right) \end{array}\right\|}^{2}}$$

where Ni is the number of incidences.

#### 2.2.2. DCS

In this section, we utilize the scattered field data to obtain the initial distribution of the dielectric constant through the DCS. This approach streamlines the CNN training process and is robust against noise interference, ensuring a more accurate estimation.

In the first step, we apply Singular Value Decomposition (SVD) to decompose the matrices $\left[G_{2}\right]$ and $\left[\begin{array}{cc} \left[G_{6}\right] & \left[G_{7}\right]\\ \left[G_{7}\right] & \left[G_{8}\right] \end{array}\right]$

(38) $$\left[G_{2}\right]=\left[U_{m}\right]\left[W_{m}\right]{\left[V_{m}\right]}^{*}$$

(39) $$\left[\begin{array}{cc} \left[G_{6}\right] & \left[G_{7}\right]\\ \left[G_{7}\right] & \left[G_{8}\right] \end{array}\right]=\left[U_{e}\right]\left[W_{e}\right]{\left[V_{e}\right]}^{*}$$

where $U_{m}$ and $U_{e}$ are the left singular vectors, while $W_{m}$ and $W_{e}$ are the diagonal matrices containing the singular values. Additionally, $V_{m}$ and $V_{e}$ are the right singular vectors. From Equations (8) and (9), it is evident that the dominant current $I_{z}^{s}$ is associated with the first $L_{m}$ row vectors of the right matrix $V_{m}$, where $L_{m}$ serves as the adjustment parameter. Similarly, from Equation (37), the dominant current $\left(\begin{array}{c} I_{x}^{s}\\ I_{y}^{s} \end{array}\right)$ is associated with the first $L_{e}$ row vectors of the right matrix $V_{e}$, where $L_{e}$ serves as the adjustment parameter.

(40) $$I_{z}^{s}=\sum_{i=1}^{L_{m}}\frac{{\left(U_{m}\right)}_{i}^{*}\cdot E_{z}^{s}}{{\left(W_{m}\right)}_{i,\;i}}{\left(V_{m}\right)}_{i},\;i=1\ldots L_{m}$$

(41) $$\left(\begin{array}{c} I_{x}^{s}\\ I_{y}^{s} \end{array}\right)=\sum_{i=1}^{L_{e}}\frac{{\left(U_{e}\right)}_{i}^{*}\cdot \left(\begin{array}{c} E_{x}^{s}\\ E_{y}^{s} \end{array}\right)}{{\left(W_{e}\right)}_{i,\;i}}{\left(V_{e}\right)}_{i},\;i=1\ldots L_{e}$$

In Equations (40) and (41), the first L singular values are considerably larger than the remaining singular values. As a result, when the measured scattered field experiences noise disturbances, the induced current remains relatively stable. The induced current can be mathematically defined as follows:

(42) $$I_{z}^{d}=I_{z}^{s}+I_{z}^{l}$$

(43) $$\left(\begin{array}{c} I_{x}^{d}\\ I_{y}^{d} \end{array}\right)=\left(\begin{array}{c} I_{x}^{s}\\ I_{y}^{s} \end{array}\right)+\left(\begin{array}{c} I_{x}^{l}\\ I_{y}^{l} \end{array}\right)$$

The low-frequency induced current is defined as follows:

(44) $$I_{z}^{l}={\overset{̿}{F}}_{z}^{l}\cdot \psi_{z}^{l}$$

(45) $$\left(\begin{array}{c} I_{x}^{l}\\ I_{y}^{l} \end{array}\right)={\overset{̿}{F}}_{xy}^{l}\cdot \left(\begin{array}{c} \psi_{x}^{l}\\ \psi_{y}^{l} \end{array}\right)$$

where ${\overset{̿}{F}}_{z}^{l}$ and ${\overset{̿}{F}}_{xy}^{l}$ denote the low-frequency Fourier transforms, while $\psi_{z}^{l}$, $\psi_{x}^{l}$ and $\psi_{y}^{l}$ represent the associated coefficients. Section 4 elaborates on the criteria for selecting the parameters $L_{m}$ and $L_{e}$.

In the second step, we apply the operator diag(τ) to both sides of Equations (32) and (33) rearrange the resulting terms to derive the equation that describes the induced current as follows:

(46) $$\left(I_{z}^{d}\right)=diag[\tau_{z}]\cdot \left[\left(E_{z}^{i}\right)+\left[G_{1}\right]\cdot \left(I_{z}^{d}\right)\right]$$

(47) $$\left(\begin{array}{c} I_{x}^{d}\\ I_{y}^{d} \end{array}\right)=diag\left[\begin{array}{cc} \left[\tau_{x}\right] & 0\\ 0 & \left[\tau_{y}\right] \end{array}\right]\cdot \left[\left(\begin{array}{c} E_{x}^{i}\\ E_{y}^{i} \end{array}\right)+\left[\begin{array}{cc} \left[G_{3}\right] & \left[G_{4}\right]\\ \left[G_{4}\right] & \left[G_{5}\right] \end{array}\right]\cdot \left(\begin{array}{c} I_{x}^{d}\\ I_{y}^{d} \end{array}\right)\right]$$

By standardizing the state and data equations, we can obtain:

(48) $$F\left(\psi_{z}^{l}\right)=\left[\frac{{\left\|\left(\left[G_{2}\right]{\overset{̿}{F}}_{z}^{l}\right)\psi_{z}^{l}+\left(\left[G_{2}\right]\left(I_{z}^{s}\right)-\left(E_{z}^{s}\right)\right)\right\|}^{2}}{{\left\|\left(E_{z}^{s}\right)\right\|}^{2}}+\frac{{\left\|\left({\overset{̿}{F}}_{z}^{l}-\left[\tau \right]\left(\left[G_{1}\right]{\overset{̿}{F}}_{z}^{l}\right)\right)\psi_{z}^{l}-\left(\left[\tau \right]\left(\left(E_{z}^{i}\right)+\left[G_{1}\right]\left(I_{z}^{s}\right)\right)-\left(I_{z}^{s}\right)\right)\right\|}^{2}}{{\left\|\left(I_{z}^{s}\right)\right\|}^{2}}\right]$$

$$F\left(\begin{array}{c} \psi_{x}^{l}\\ \psi_{y}^{l} \end{array}\right)=\left[\frac{{\left\|\left(\left[\begin{array}{cc} \left[G_{6}\right] & \left[G_{7}\right]\\ \left[G_{7}\right] & \left[G_{8}\right] \end{array}\right]{\overset{̿}{F}}_{xy}^{l}\right)\left(\begin{array}{c} \psi_{x}^{l}\\ \psi_{y}^{l} \end{array}\right)+\left(\left[\begin{array}{cc} \left[G_{6}\right] & \left[G_{7}\right]\\ \left[G_{7}\right] & \left[G_{8}\right] \end{array}\right]\left(\begin{array}{c} I_{x}^{s}\\ I_{y}^{s} \end{array}\right)-\left(\begin{array}{c} E_{x}^{s}\\ E_{y}^{s} \end{array}\right)\right)\right\|}^{2}}{{\left\|\left(\begin{array}{c} E_{x}^{s}\\ E_{y}^{s} \end{array}\right)\right\|}^{2}}+\right.$$

(49) $$\frac{{\left\|\left({\overset{̿}{F}}_{xy}^{l}-\left[\begin{array}{cc} \left[\tau_{x}\right] & 0\\ 0 & \left[\tau_{y}\right] \end{array}\right]\left(\left[\begin{array}{cc} \left[G_{3}\right] & \left[G_{4}\right]\\ \left[G_{4}\right] & \left[G_{5}\right] \end{array}\right]{\overset{̿}{F}}_{xy}^{l}\right)\right)\left(\begin{array}{c} \psi_{x}^{l}\\ \psi_{y}^{l} \end{array}\right)-\left(\left[\begin{array}{cc} \left[\tau_{x}\right] & 0\\ 0 & \left[\tau_{y}\right] \end{array}\right]\left(\left(\begin{array}{c} E_{x}^{i}\\ E_{y}^{i} \end{array}\right)+\left[\begin{array}{cc} \left[G_{3}\right] & \left[G_{4}\right]\\ \left[G_{4}\right] & \left[G_{5}\right] \end{array}\right]\left(\begin{array}{c} I_{x}^{s}\\ I_{y}^{s} \end{array}\right)\right)-\left(\begin{array}{c} I_{x}^{s}\\ I_{y}^{s} \end{array}\right)\right)\right\|}^{2}}{{\left\|\left(\begin{array}{c} I_{x}^{s}\\ I_{y}^{s} \end{array}\right)\right\|}^{2}}$$

To avoid the need for matrix inversion, we propose an approximate analytical solution for $\psi^{l}$. This method is designed to minimize the cost functions (8) and (9) in the direction of the gradient $\bar{\rho }$.

(50) $$\psi_{z}^{l}=\beta_{z}{\bar{\rho }}_{z}$$

(51) $$\left(\begin{array}{c} \psi_{x}^{l}\\ \psi_{y}^{l} \end{array}\right)=\left(\begin{array}{c} \beta_{x}{\bar{\rho }}_{x}\\ \beta_{y}{\bar{\rho }}_{y} \end{array}\right)$$

The gradient direction function is defined as follows:

(52) $$F^{\prime }\left(\psi_{z}^{l}=0\right)={\bar{\rho }}_{z}=\frac{{\left(\left[G_{2}\right]{\overset{̿}{F}}^{l}\right)}^{H}\left(\left[G_{2}\right]\left(I_{z}^{s}\right)-\left(E_{z}^{s}\right)\right)}{{\left\|\left(E_{z}^{s}\right)\right\|}^{2}}+\frac{-{\left({\overset{̿}{F}}^{l}-\left[\tau_{z}\right]\left(\left[G_{1}\right]{\overset{̿}{F}}^{l}\right)\right)}^{H}\left(\left[\tau_{z}\right]\left(\left(E_{z}^{i}\right)+\left[G_{1}\right]\left(I_{z}^{s}\right)\right)-\left(I_{z}^{s}\right)\right)}{{\left\|I_{z}^{s}\right\|}^{2}}$$

$$F^{\prime }\left(\begin{array}{c} \psi_{x}^{l}\\ \psi_{y}^{l} \end{array}=0\right)=\left(\begin{array}{c} {\bar{\rho }}_{x}\\ {\bar{\rho }}_{y} \end{array}\right)=\left[\frac{{\left(\left[\begin{array}{cc} \left[G_{6}\right] & \left[G_{7}\right]\\ \left[G_{7}\right] & \left[G_{8}\right] \end{array}\right]{\overset{̿}{F}}_{xy}^{l}\right)}^{H}\left(\left(\left[\begin{array}{cc} \left[G_{6}\right] & \left[G_{7}\right]\\ \left[G_{7}\right] & \left[G_{8}\right] \end{array}\right]\left(\begin{array}{c} I_{x}^{s}\\ I_{y}^{s} \end{array}\right)\right)-\left(\begin{array}{c} E_{x}^{s}\\ E_{y}^{s} \end{array}\right)\right)}{{\left\|\left(\begin{array}{c} E_{x}^{s}\\ E_{y}^{s} \end{array}\right)\right\|}^{2}}+\right.$$

(53) $$\left.\frac{{-\left({\overset{̿}{F}}_{xy}^{l}-\left[\begin{array}{cc} \left[\tau_{x}\right] & 0\\ 0 & \left[\tau_{y}\right] \end{array}\right]\left(\left[\begin{array}{cc} \left[G_{3}\right] & \left[G_{4}\right]\\ \left[G_{4}\right] & \left[G_{5}\right] \end{array}\right]{\overset{̿}{F}}_{xy}^{l}\right)\right)}^{H}\left(\left[\begin{array}{cc} \left[\tau_{x}\right] & 0\\ 0 & \left[\tau_{y}\right] \end{array}\right]\left(\left(\begin{array}{c} E_{x}^{i}\\ E_{y}^{i} \end{array}\right)+\left[\begin{array}{cc} \left[G_{3}\right] & \left[G_{4}\right]\\ \left[G_{4}\right] & \left[G_{5}\right] \end{array}\right]\left(\begin{array}{c} I_{x}^{s}\\ I_{y}^{s} \end{array}\right)\right)-\left(\begin{array}{c} I_{x}^{s}\\ I_{y}^{s} \end{array}\right)\right)}{{\left\|\left(\begin{array}{c} I_{x}^{s}\\ I_{y}^{s} \end{array}\right)\right\|}^{2}}\right]$$

The minimizer function is defined as follows:

(54) $$\beta_{z}=\frac{\begin{array}{ccc} -\frac{{\left(\left[G_{2}\right]{\overset{̿}{F}}_{z}^{l}{\bar{\rho }}_{z}\right)}^{H}\left(\left[G_{2}\right]\left(I_{z}^{s}\right)-\left(E_{z}^{s}\right)\right)}{{\left\|\left(E_{z}^{s}\right)\right\|}^{2}} & + & \frac{{\left(\left({\overset{̿}{F}}_{z}^{l}-\left[\tau \right]\left(\left[G_{1}\right]{\overset{̿}{F}}_{z}^{l}\right)\right){\bar{\rho }}_{z}\right)}^{H}\left(\left[\tau \right]\left(\left(E_{z}^{i}\right)+\left[G_{1}\right]\left(I_{z}^{s}\right)\right)-\left(I_{z}^{s}\right)\right)}{{\left\|\left(I_{z}^{s}\right)\right\|}^{2}} \end{array}}{\begin{array}{ccc} \frac{{\left\|\left[G_{2}\right]{\overset{̿}{F}}_{z}^{l}{\bar{\rho }}_{z}\right\|}^{2}}{{\left\|\left(E_{z}^{s}\right)\right\|}^{2}} & + & \frac{{\left\|\left({\overset{̿}{F}}_{z}^{l}-\left[\tau \right]\left(\left[G_{1}\right]{\overset{̿}{F}}_{z}^{l}\right)\right){\bar{\rho }}_{z}\right\|}^{2}}{{\left\|\left(I_{z}^{s}\right)\right\|}^{2}} \end{array}}$$

$$\left(\begin{array}{c} \beta_{x}\\ \beta_{y} \end{array}\right)=\frac{-\frac{{\left(\left[\begin{array}{cc} \left[G_{6}\right] & \left[G_{7}\right]\\ \left[G_{7}\right] & \left[G_{8}\right] \end{array}\right]{\overset{̿}{F}}_{xy}^{l}\left(\begin{array}{c} {\bar{\rho }}_{x}\\ {\bar{\rho }}_{y} \end{array}\right)\right)}^{H}\left(\left[\begin{array}{cc} \left[G_{6}\right] & \left[G_{7}\right]\\ \left[G_{7}\right] & \left[G_{8}\right] \end{array}\right]\left(\begin{array}{c} I_{x}^{s}\\ I_{y}^{s} \end{array}\right)-\left(\begin{array}{c} {\bar{E}}_{x}^{s}\\ {\bar{E}}_{y}^{s} \end{array}\right)\right)}{{\left\|\left(\begin{array}{c} E_{x}^{s}\\ E_{y}^{s} \end{array}\right)\right\|}^{2}}}{\frac{{\left\|\left[\begin{array}{cc} \left[G_{6}\right] & \left[G_{7}\right]\\ \left[G_{7}\right] & \left[G_{8}\right] \end{array}\right]{\overset{̿}{F}}_{xy}^{l}\left(\begin{array}{c} {\bar{\rho }}_{x}\\ {\bar{\rho }}_{y} \end{array}\right)\right\|}^{2}}{{\left\|\left(\begin{array}{c} E_{x}^{s}\\ E_{y}^{s} \end{array}\right)\right\|}^{2}}}+$$

(55) $$\frac{\frac{{{\left(\left({\overset{̿}{F}}_{xy}^{l}-\left[\begin{array}{cc} \left[\tau_{x}\right] & 0\\ 0 & \left[\tau_{y}\right] \end{array}\right]\left(\left[\begin{array}{cc} \left[G_{3}\right] & \left[G_{4}\right]\\ \left[G_{4}\right] & \left[G_{5}\right] \end{array}\right]{\overset{̿}{F}}_{xy}^{l}\right)\right)\left(\begin{array}{c} {\bar{\rho }}_{x}\\ {\bar{\rho }}_{y} \end{array}\right)\right)}^{H}\left(\left[\begin{array}{cc} \left[\tau_{x}\right] & 0\\ 0 & \left[\tau_{y}\right] \end{array}\right]\left(\left(\begin{array}{c} E_{x}^{i}\\ E_{y}^{i} \end{array}\right)+\left[\begin{array}{cc} \left[G_{3}\right] & \left[G_{4}\right]\\ \left[G_{4}\right] & \left[G_{5}\right] \end{array}\right]\left(\begin{array}{c} I_{x}^{s}\\ I_{y}^{s} \end{array}\right)\right)-\left(\begin{array}{c} I_{x}^{s}\\ I_{y}^{s} \end{array}\right)\right)}^{H}}{{\left\|\left(\begin{array}{c} I_{x}^{s}\\ I_{y}^{s} \end{array}\right)\right\|}^{2}}}{\frac{{\left\|\left({\overset{̿}{F}}_{xy}^{l}-\left[\begin{array}{cc} \left[\tau_{x}\right] & 0\\ 0 & \left[\tau_{y}\right] \end{array}\right]\left(\left[\begin{array}{cc} \left[G_{3}\right] & \left[G_{4}\right]\\ \left[G_{4}\right] & \left[G_{5}\right] \end{array}\right]{\overset{̿}{F}}_{xy}^{l}\right)\right)\left(\begin{array}{c} {\bar{\rho }}_{x}\\ {\bar{\rho }}_{y} \end{array}\right)\right\|}^{2}}{{\left\|\left(\begin{array}{c} I_{x}^{s}\\ I_{y}^{s} \end{array}\right)\right\|}^{2}}}$$

The final step involves calculating the contrast $\left[\tau_{p}^{d}\right]$ of the DCS using $I^{d}$. We update the total electric field function $E_{p}^{d}$ for the *p*-th incidence DCS as follows:

(56) $$\left(E_{z,p}^{d}\right)=\left(E_{z,p}^{i}\right)+\left[G_{1}\right]\left(I_{z,p}^{d}\right)$$

(57) $$\left(\begin{array}{c} E_{x,p}^{d}\\ E_{y,p}^{d} \end{array}\right)=\left(\begin{array}{c} E_{x}^{i}\\ E_{y}^{i} \end{array}\right)+\left[\begin{array}{cc} \left[G_{3}\right] & \left[G_{4}\right]\\ \left[G_{4}\right] & \left[G_{5}\right] \end{array}\right]\left(\begin{array}{c} I_{x,p}^{d}\\ I_{y,p}^{d} \end{array}\right)$$

Based on the definitions of (53) and (54), the *n*-th component of the contrast $\tau_{p}^{d}$ for the *p*-th incidence

(58) $$I_{z,p}^{d}=diag\left({\tau_{z}}_{p}^{d}\right)\cdot \left(E_{z,p}^{d}\right)$$

(59) $$\left(\begin{array}{c} I_{x,p}^{d}\\ I_{y,p}^{d} \end{array}\right)=diag\left[\begin{array}{cc} \left[{\tau_{x}}_{p}^{d}\right] & 0\\ 0 & \left[{\tau_{y}}_{p}^{d}\right] \end{array}\right]\cdot \left(\begin{array}{c} E_{x,p}^{d}\\ E_{y,p}^{d} \end{array}\right)$$

can be calculated:

(60) $${\tau_{z}}_{p}^{d}(n)=\frac{I_{z,p}^{d}\left(n\right){\cdot \left(E_{z,p}^{d}\left(n\right)\right)}^{*}}{{\left\|E_{z,p}^{d}\left(n\right)\right\|}^{2}}$$

(61) $$\left[\begin{array}{cc} \left[{\tau_{x}}_{p}^{d}\right] & 0\\ 0 & \left[{\tau_{y}}_{p}^{d}\right] \end{array}\right]\left(n\right)=\frac{\left(\begin{array}{c} I_{x,p}^{d}\\ I_{y,p}^{d} \end{array}\right)\left(n\right){\cdot \left(\left(\begin{array}{c} E_{x,p}^{d}\\ E_{y,p}^{d} \end{array}\right)\left(n\right)\right)}^{*}}{{\left\|\left(\begin{array}{c} E_{x,p}^{d}\\ E_{y,p}^{d} \end{array}\right)\left(n\right)\right\|}^{2}}$$

## 3. Convolutional Neural Network

**Figure 3:**
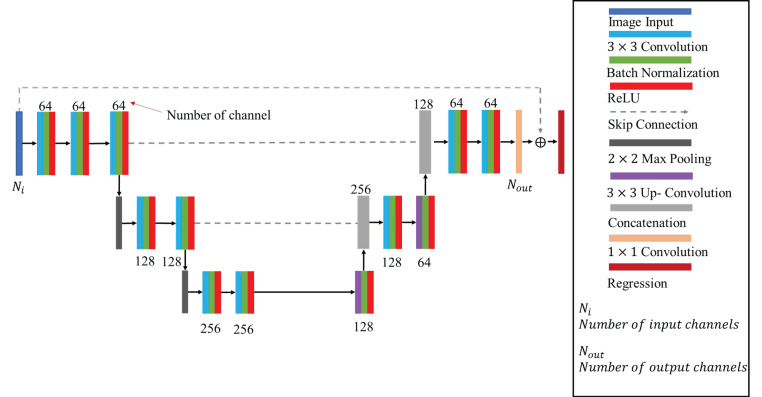
Convolutional Neural Network architecture.

AI technology is evolving at an impressive pace, demonstrating broad applicability across various domains such as speech recognition, image processing, and autonomous vehicles. In addition to these fields, the integration of AI methodologies into electromagnetic imaging techniques has become increasingly prominent, particularly in conjunction with CNNs, Deep Convolutional Neural Networks (DCNNs), and Generative Adversarial Networks (GANs). This paper implements a CNN architecture specifically tailored to tackle the inverse scattering problem in a half-space, as depicted in Figure 3. The architecture is composed of two main components: a contraction network on the left and an expansion network on the right. On the contraction side, we sequentially arrange layers that include 3×3 convolutional layers, normalization layers, ReLU (Rectified Linear Unit) activations, and 2×2 pooling layers. The expansion network is structured to mirror the contraction network, utilizing 3×3 deconvolutional layers, normalization layers, and a ReLU activation layer. The architecture culminates in a 1×1 convolutional layer that acts as a fully connected layer. The resultant averaged output is then forwarded to a regression layer to evaluate the error associated with the dielectric coefficient distribution. The cost function can be defined as follows:

(62) $$\begin{array}{c} argmin\\ A_{i1},i \end{array}:\sum_{N=1}^{N_{t}}f\left(A_{i1}\left(\varepsilon_{z}^{\alpha }\right),\;\varepsilon_{z}\right)+Q_{1}\left(i\right)$$

(63) $$\begin{array}{c} argmin\\ A_{i2},i \end{array}:\sum_{N=1}^{N_{t}}f\left(A_{i2}\left(\begin{array}{c} \varepsilon_{x}^{\alpha }\\ \varepsilon_{y}^{\alpha } \end{array}\right),\;\left(\begin{array}{c} \varepsilon_{x}\\ \varepsilon_{y} \end{array}\right)\right)+Q_{2}\left(i\right)$$

where $A_{i1}$ and $A_{i2}$ indicate the parameters of the neural network architecture. f signifies the difference between the image generated by the CNN and the ground truth. $\varepsilon^{\alpha }$ refers to the approximate permittivity coefficient used in the model. $Q_{1\left(i\right)}$ and $Q_{2\left(i\right)}$ are regularization functions applied to mitigate overfitting and enhance model generalization, where *Q*_1_(*i*) is an L2-norm regularization function of z-axis permittivity and *Q*_2_(*i*) is an L2-norm regularization function of x-axis permittivity, respectively. In addition to helping mitigate overfitting, it also improves the convergence of the optimization process.

As CNNs are widely recognized for their superior performance in image processing tasks, we opted to employ a CNN architecture to reconstruct electromagnetic images in this paper. The rationale behind selecting CNNs is multi-faceted, involving the following factors:

1. Skip connections between the input and output layers of a CNN play a critical role in addressing the vanishing gradient problem, ensuring a more consistent gradient flow during backpropagation.
2. Down-sampling in the contraction pathway of a CNN expands the receptive data, which improves the network’s ability to make accurate pixel-level predictions in the output.
3. Batch normalization, an integral component of the CNN architecture, mitigates internal covariate shift, accelerates convergence, and reduces sensitivity to parameter initialization and gradient instability.

In the next section, we explore the reconstruction performance of the BPS and the DCS in a half-space environment using a CNN. Our analysis emphasizes the noise resilience of both methods and examines how the material properties of buried objects affect reconstruction accuracy, providing insights into their respective strengths and limitations.

## 4. Numerical Result

In this study, we explore the dielectric constant distributions of uniaxial objects buried in soil at a depth of 2 m. In other words, we assume that $\varepsilon_{x}$ is equivalent to $\varepsilon_{y}$. A configuration comprising 32 receivers spanning from θ= 195° to 350°, with a radius of distance set at 3 m and 32 transmitters ranging from $\emptyset_{1}=$ −80° to 80° at 5° intervals, are employed in our simulation. To emulate real-world conditions, 5%, 10%, 15%, and 20% Gaussian noise are added. We consider scatterers characterized by 10 distinct dielectric constant profiles, positioned randomly across 50 predefined locations within the measurement area. This means that we generate a dataset comprising 16,000 images for each configuration (calculated as 10×50×32). This dataset is subsequently partitioned into 80% for training and 20% for testing purposes. We use Stochastic Gradient Descent with Momentum (SGDM) to train the CNN. The training configuration includes a momentum factor of 0.99, a learning rate ranging from $10^{-4}$ to $10^{-5}$, and a maximum of 40 epochs. The input of the U-Net is derived from both the BPS and DCS methods to estimate the preliminary dielectric distributions. The efficacy of the two approaches is assessed through a comparison of the U-Net reconstruction outcomes. We calculate the Root Mean Square Error (RMSE) and the Structural Similarity Index Measure (SSIM) using the formulations in Equations (59) and (60), respectively. These metrics are employed to assess the performance of each scenario comprehensively.

(64) $$RMSE=\frac{1}{M_{t}}\sum_{i=1}^{M_{t}}\frac{{\left\|{\overset{̿}{\varepsilon }}_{r}-{\overset{̿}{\varepsilon }}_{r}^{r}\right\|}_{F}}{{\left\|{\overset{̿}{\varepsilon }}_{r}\right\|}_{F}}$$

(65) $$SSIM=\frac{\left(2\mu_{\tilde{y}}\mu_{y}+C_{1}\right)\left(2\sigma_{\tilde{y}y}+C_{2}\right)}{\left(\mu_{\tilde{y}}^{2}+\mu_{y}^{2}+C_{1}\right)\left(\sigma_{\tilde{y}}^{2}+\sigma_{y}^{2}+C_{2}\right)}$$

where ${\overset{̿}{\varepsilon }}_{r}$ and ${\overset{̿}{\varepsilon }}_{r}^{r}$ refer to the actual and reconstructed profiles of the relative permittivity, respectively. $M_{t}$ represents the total number of experimental tests performed, while F signifies the Frobenius norm. $\tilde{y}$ and y represent the reconstructed and true relative permittivity profiles, respectively. $\mu_{y}$ denotes the mean of the reconstructed profile y, while $\sigma_{\tilde{y}}^{2}$ captures the variance, and $\sigma_{\tilde{y}y}$ signifies the covariance between $\tilde{y}$ and y. To address potential issues with zero denominators in the calculations, we introduce two small constraints, $C_{1}={\left(K_{1}D\right)}^{2}$ and $C_{2}={\left(K_{2}D\right)}^{2}$, where $K_{1}=0.01$ and $K_{2}=0.03$ serve as hyperparameters. Here, D represents the dynamic range of the pixel intensity values in the target image y.

### 4.1. Relative Permittivity Between 3.5 and 4

In this case, we define the permittivity distribution within a range of 3.5 to 4. We examine a scenario involving 10 scatterers, each with distinct permittivity profiles, positioned at 50 different locations throughout the designated measurement area. To simulate a realistic environment, we introduce 20% Gaussian noise into the scattered field data. The dielectric coefficient distribution is estimated using both the BPS and DCS techniques. These estimates are then input into a CNN to reconstruct electromagnetic images. Finally, we conduct a comparative analysis of the reconstruction outcomes from the two different sets of input data.

**Figure 4:**
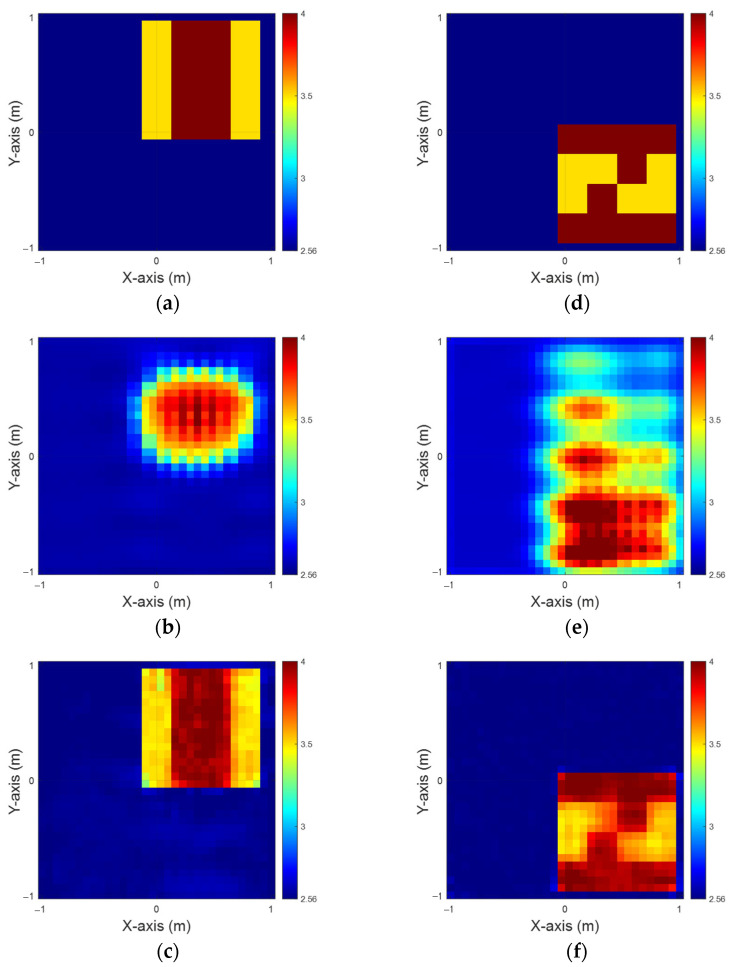
Relative permittivity between 3.5 and 4. (**a**) Ground truth for $\varepsilon_{z}.$ (**b**) BPS reconstruction outcome for $\varepsilon_{z}.$ (**c**) DCS reconstruction outcome for $\varepsilon_{z}.$ (**d**) Ground truth for $\varepsilon_{x}.$ (**e**) BPS reconstruction outcome for $\varepsilon_{x}.$ (**f**) DCS reconstruction outcome for $\varepsilon_{x}$.

Figure 4a,d illustrate the ground truth for the $\varepsilon_{z}$ and $\varepsilon_{x}$ configurations, respectively. Figure 4b,e present the BPS reconstruction outcomes. Figure 4c,f showcase the DCS reconstruction results for the $\varepsilon_{z}$ and $\varepsilon_{x}$ cases, respectively. The findings indicate that when an object is buried within a half-space medium, the scattering of incident waves significantly impairs the reconstruction accuracy. This study demonstrates that despite employing two distinct input configurations and a well-trained CNN, the BPS method is limited to approximating the object’s location. In contrast, the DCS approach achieves a more accurate determination of both the position and distribution of the buried materials. Additionally, Table 1 provides the Root Mean Square Error (RMSE) and Structural Similarity Index Measure (SSIM) metrics for this case.

### 4.2. Relative Permittivity Between 4 and 4.5

In this case, the permittivity distribution is defined within the range of 4 to 4.5. We consider a scenario with 10 scatterers, each exhibiting unique permittivity profiles, positioned at 50 distinct locations across the designated measurement area. To mimic realistic conditions, 5% Gaussian noise is added to the scattered field data. The dielectric coefficient distribution is estimated using both the BPS and DCS methods. These estimates serve as the input for CNN to reconstruct electromagnetic images. Finally, we perform a comparative analysis of the reconstruction results obtained from the two different input datasets.

**Figure 5:**
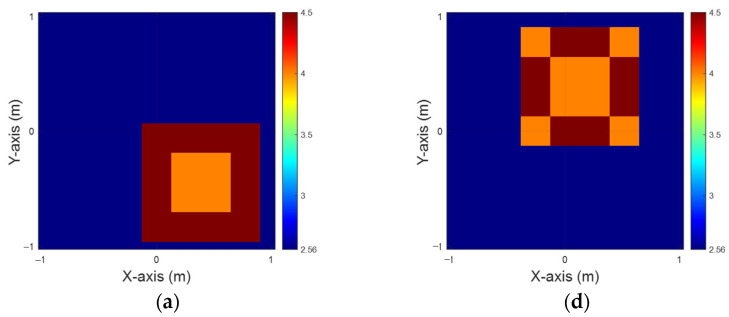

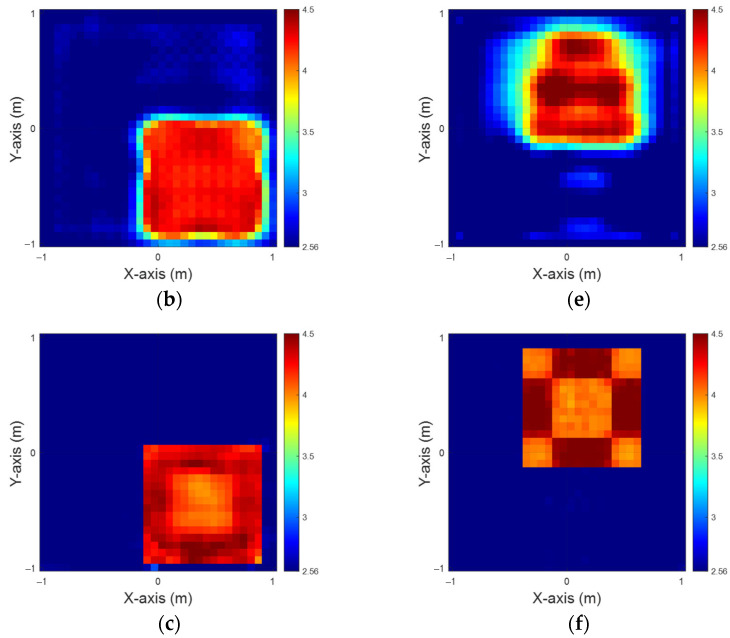
Relative permittivity between 4 and 4.5. (**a**) Ground truth for $\varepsilon_{z}.$ (**b**) BPS reconstruction outcome for $\varepsilon_{z}.$ (**c**) DCS reconstruction outcome for $\varepsilon_{z}.$ (**d**) Ground truth for $\varepsilon_{x}.$ (**e**) BPS reconstruction outcome for $\varepsilon_{x}.$ (**f**) DCS reconstruction outcome for $\varepsilon_{x}$.

Figure 5a,d show the ground truth configurations for $\varepsilon_{z}$ and $\varepsilon_{x}$, respectively. Figure 5b,e display the reconstruction results obtained using the BPS method, while Figure 5c,f present the DCS reconstruction outcomes for the $\varepsilon_{z}$ and $\varepsilon_{x}$ cases, respectively. The results reveal that the presence of an object buried in a half-space medium causes significant scattering of incident waves, adversely affecting reconstruction accuracy. In addition, the nonlinearity increases proportionally with the dielectric constant of the embedded object, posing significant challenges to reconstructing accurate electromagnetic images. Likewise in case A, despite utilizing two distinct input configurations and a well-trained CNN, the BPS method is limited to approximating the object’s location. In contrast, the DCS method demonstrates superior performance, providing more accurate estimates of both the position and distribution of buried materials. Table 2 summarizes the RMSE and SSIM metrics for this scenario.

### 4.3. Relative Permittivity Between 4.5 and 5 Using the Model in Section 4.2

**Figure 6:**
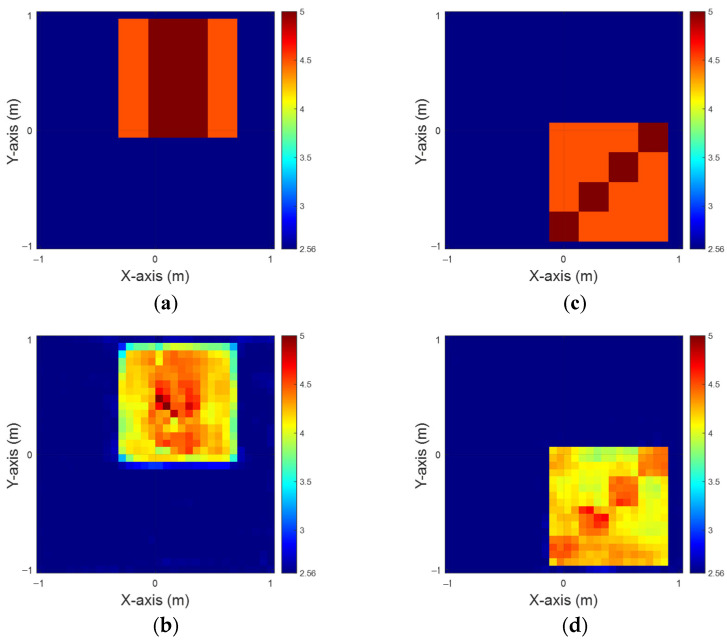
Relative permittivity between 4.5 and 5 using Section 4.2 model. (**a**) ground truth for $\varepsilon_{z}$ (**b**) ground truth for $\varepsilon_{x}$ (**c**) DCS reconstruction outcome for $\varepsilon_{z}$ (**d**) DCS reconstruction outcome for $\varepsilon_{x}$.

In this case, the permittivity distribution is defined within the range of 4.5 to 5. We consider a scenario with 10 scatterers, each exhibiting unique permittivity profiles, positioned at 50 distinct locations across the designated measurement area. To mimic realistic conditions, 5% Gaussian noise is added to the scattered field data. The dielectric coefficient distribution is estimated using the DCS method. The estimates serve as inputs to the CNN for image reconstruction. In practical scenarios, noise interference is prevalent and has a significant impact on reconstruction outcomes. We use the model in Section 4.2 to reconstruct the relative permittivity. Figure 6a,c are the ground truth for $\varepsilon_{z}$ and $\varepsilon_{x}$, respectively. Figure 6b,d are the DCS reconstruction results of $\varepsilon_{z}$ and $\varepsilon_{x}$ using the Section 4.2 model, respectively. The numerical results show that our proposed method has good generalization ability. Table 3 summarizes the RMSE and SSIM metrics for this scenario.

## 5. Conclusions

We propose a novel method for the electromagnetic imaging of uniaxial objects buried in a half-space using CNNs. Measurement constraints make it difficult to gather sufficient information for improved reconstruction. We use the TM and TE waves to illuminate a uniaxial object, receive the scattered fields, and calculate the estimated permittivity distribution using the BPS and the DCS. The results show that the DCS, when processed through CNNs, provides a more accurate dielectric constant than the BPS. The TE case is more complex due to the vector nature of the TE electric field. Traditional algorithms are computationally expensive, whereas CNNs provide benefits like lower costs and faster processing in under one second, making it effective for electromagnetic inverse scattering problems. Compared to the BPS, a major drawback of the DCS is the significant computational resources and time required for estimating the initial dielectric constant distribution. However, when combined with a CNN, this approach produces impressive reconstruction results. It effectively tackles nonlinear phenomena and exhibits robust resistance to noise.

Future research will investigate the relationship between anisotropic parameters across various real-world objects and explore the potential of applying constraints to biaxial anisotropic parameters. There are promising directions in AI applications for electromagnetic imaging, such as using generative adversarial network and segmenting scatterers for high-resolution reconstruction.

## Figures and Tables

**Table 1:** RMSE and SSIM for $\varepsilon_{z}$ and $\varepsilon_{x}$ for relative permittivity between 3.5 and 4.

Performance	$\varepsilon_{z}$		$\varepsilon_{x}$	
	BPS	DCS	BPS	DCS
RMSE	12.03%	1.43%	16.37%	1.25%
SSIM	57.32%	95.9%	29.19%	99.02%

**Table 2:** RMSE and SSIM for $\varepsilon_{z}$ and $\varepsilon_{x}$ for relative permittivity between 4 and 4.5.

Performance	$\varepsilon_{z}$		$\varepsilon_{x}$	
	BPS	DCS	BPS	DCS
RMSE	7.8%	3.12%	9.72%	0.82%
SSIM	81.02%	97.46%	78.76%	99.62%

**Table 3:** RMSE and SSIM for $\varepsilon_{z}$ and $\varepsilon_{x}$ for relative permittivity between 4.5 and 5.

Performance	$\varepsilon_{z}$	$\varepsilon_{x}$
RMSE	9.04%	8.02%
SSIM	91.28%	96.04%

## Data Availability

The original contributions presented in this study are included in the article. Further inquiries can be directed to the corresponding author.
